# Association Between Interleukin 35 Gene Single Nucleotide Polymorphisms and the Uveitis Immune Status in a Chinese Han Population

**DOI:** 10.3389/fimmu.2021.758554

**Published:** 2021-12-07

**Authors:** Meng Feng, Shuping Zhou, Tong Liu, Yong Yu, Qinghong Su, Xiaofan Li, Min Zhang, Xiao Xie, Tingting Liu, Wei Lin

**Affiliations:** ^1^ School of Basic Medicine, Shandong First Medical University & Shandong Academy of Medical Sciences, Jinan, China; ^2^ Departments of Medicine, Tibet Nationalities University, Xianyang, China; ^3^ Ophthalmology Department, Eye Hospital of Shandong First Medical University (Shandong Eye Hospital), Jinan, China; ^4^ State Key Laboratory Cultivation Base, Shandong Provincial Key Laboratory of Ophthalmology, Shandong Eye Institute, Shandong First Medical University & Shandong Academy of Medical Sciences, Qingdao, China; ^5^ The First Clinical Medical College, Shandong University of Chinese Medicine, Jinan, China; ^6^ School of Ophthalmology, Shandong First Medical University, Jinan, China

**Keywords:** interleukin-35, regulatory B cells, interleukin-12p35, EBI3, Behçet’s syndrome, Vogt–Koyanagi–Harada syndrome, gene single nucleotide polymorphisms, autoimmune diseases

## Abstract

Autoimmune uveitis is characterized by immune disorders of the eyes and the whole body and is often recurrent in young adults, but its pathogenesis is still unclear. IL-35 is an essential regulatory factor in many autoimmune diseases, which is produced by Breg cells and can induce Breg cells to regulate the immune response. The relationship between the expression and gene polymorphism of IL-35 and the immune status of patients with autoimmune uveitis has not been reported. The peripheral blood of the subjects was collected from patients with Behçet’s Disease (BD) and those with Vogt–Koyanagi–Harada (VKH) syndrome. The percentage of immune cell subsets including B cells, DC, and T cells, and the expression of IL-35 in serum of these two kinds of disease were analyzed. And then, the associations between seven IL-35 single nucleotide polymorphism (SNP) sites and disease susceptibility, the immune status, the clinical characteristics, and the serum IL-35 levels were analyzed. Our results showed that the percentage of Breg cells was significantly decreased in the blood of patients with VKH syndrome compared to that of healthy controls. The levels of IL-35 in the serum of patients with VKH syndrome or BD patients were not changed significantly, compared to that of healthy controls. Furthermore, the associations between two subunits of IL-35 (IL-12p35 and EBI3) and BD or VKH patients were analyzed. We found that there was an association between the EBI3 rs428253 and the occurrence of BD. There was an association between the IL-12p35 rs2243131 and the low level of Breg cell of VKH patients. In addition, there were associations between the polymorphisms of EBI3 rs4740 and the occurrence of headache and tinnitus of VKH patients, respectively. And the genotype frequency of IL-12p35 rs2243115 was related to the concentration of serum IL-35 in patients with VKH syndrome. Thus, the specific SNP sites change of IL-35 were correlated to the immune disorders in uveitis. And they may also play a guiding role in the occurrence of clinical symptoms in patients with uveitis, especially for VKH syndrome.

## Introduction

Uveitis is an autoimmune inflammatory disease that severely impairs visual function (1). Behçet’s disease (BD) and Vogt–Koyanagi–Harada (VKH) syndrome are common types of diffuse panuveitis. They usually manifest as chronic and recurrent, which affects multiple organs and systems (2–5). And in the early stage, BD patients often take dental ulcer and ophthalmia as their initial symptoms, while VKH patients are usually accompanied by headache, hearing loss, alopecia, and so on (6, 7). The disorder of the immune system is a key factor for the recurrence of the disease and visual impairment (8–10). However, the cause of the disease is not clear. It had been reported that abnormal autoreactive B cells, activated DC cells, and activated T cells increase in autoimmune uveitis (AIU), and the ratio of T helper type 17 (Th17)/regulatory T cell (Treg) cell was also increased (11, 12). Lacking immunosuppressive molecules or regulatory cells may be the cause of immune over-activation. In experimental autoimmune uveitis (EAU), regulatory B (Breg) cells secreting IL-35 suppress intraocular inflammation by inducing expansion of IL-10-producing and IL-35-producing regulatory B (Breg) and regulatory T (Treg) cells. These indicate that Breg cells orchestrate an immunosuppressive milieu in autoimmune diseases (13, 14). Yet, the statuses of Breg cells in patients with uveitis have not been thoroughly investigated, and the possible factors affecting the Breg cells in uveitis might be an important pathogenic factor for this disease.

Breg cells is a class of B cell subsets with a negative regulatory immune response (15). It had been found that Breg cells can exert their inhibitory effects with different mechanisms in different mice models of autoimmune diseases (16), such as experimental autoimmune encephalomyelitis (EAE), systemic lupus erythematosus (SLE), and uveitis (13, 17, 18). Emerging evidence suggests a potent regulatory function of IL-35 and IL-10 in orchestrating autoreactive Breg cells responses and reveals significantly impaired IL-10/IL-35-producing capacity in Breg cells upon autoimmune disease progression (15, 19). As a member of the IL-12 family, IL-35 comprises two subunits: IL-12p35 and EBI3 (20, 21). IL-12p35 induces Breg cells to secrete IL-10/IL-35, promote Breg cells expansion, stimulate Treg effects, and induce immune tolerance (13, 14). Although IL-35 participates in the proliferation and expansion of Breg cells, it also can effectively alleviate and inhibit the development of autoimmune diseases (22, 23). As previously mentioned, IL-35 induces the expression of IL-10+/IL-35+ Breg, to alleviate EAU (14). However, the status of IL-35 in patients with uveitis is still not clear.

Studying genetic susceptibility is a hot issue to explore the pathogenesis of diseases in recent years (24–26). Some studies have confirmed that there were significant associations between the SNP sites (SNPs) of IL-35 and the occurrence of certain clinical manifestations in patients with autoimmune diseases, for example, IL-35 rs4740 with patients with SLE in the Chinese Han population (24). But it is not clear whether the SNPs of IL-35 are related to the occurrence of BD or VKH.

Therefore, in our study, we separately analyzed the clinical symptoms of patients with BD and those with VKH syndrome in the Chinese Han population and the correlation between IL-35 cytokine levels and these diseases. In addition, we focused on the relationship between the SNPs of IL-35 (including its subunits IL-12p35 and EBI3) and disease susceptibility, immune statuses with BD or VKH patients. Our results shown that there were associations between the polymorphisms of EBI3 rs4740 and the occurrence of headache and tinnitus of VKH patients, respectively. The genotype frequency of IL-12p35 rs2243115 was related to the concentration of serum IL-35 in patients with VKH syndrome. In particular, the polymorphisms of IL-12p35 rs2243131 was related to Breg cells disorder in VKH patients. There was an association between EBI3 rs428253 and the occurrence of BD. Considering these, uveitis may result from the interaction of various factors between the genetic and immune environment, which may provide a new basis for its diagnosis and treatment.

## Materials and Methods

### Subjects

All subjects involved in our study have been recruited from the Shandong Eye Hospital and the Shandong Qilu Hospital Laboratory Department in 2020-2021, including 11 patients with BD, 21 patients with VKH syndrome, and 48 normal healthy people, all of whom were Chinese Han people. All patients with BD met the criteria established by the International Panel on BD (27, 28). All patients with VKH syndrome met the standard set by the international research group (29, 30). All patients were either not treated or stopped taking immunosuppressive drugs at least six months before blood samples were taken. Meanwhile, all healthy controls met the following inclusion criteria: no family history of BD syndrome; no family history of VKH syndrome, no history of autoimmune diseases and severe systemic diseases, no blood relationship with other subjects, and Chinese Han population. The informed consent of all participants was obtained, and the demographic data are consistent with the clinical characteristics. The eyes were analyzed by fundus photography and optical coherence tomography angiography (OCTA) to indicate any eyes lesions, OCTA was performed using AngioVue (Optovue, Fremont, California, USA).

### Flow Cytometry

Two milliliters of heparinized venous blood were obtained from patients with uveitis and healthy controls. Human peripheral blood mononuclear cells (PBMCs) were collected at the buffy coat layer using a Human Ficoll-Hypaque (Pharmacia) gradient and then washed twice with phosphate-buffered saline (Genview, cat#: GS3101) to remove red blood cells. Phenotypic analysis of fresh PBMCs was conducted by flow cytometry. Fluorescent antibodies of human CD45 (clone 2D1), human CD3 (clone HIT3a), human CD4 (clone OKT4), human CD8 (clone OKT8), human CD19 (clone HIB19), human CD1d (clone 51.1), human CD5 (clone UCHT2), human CD56 (clone HCD56), human CD14 (clone S18004B), human CD11c (clone Bu15), human HLA-DR (clone L243) were obtained. Furthermore, we used flow cytometry to analyze the expression of immune cells in healthy controls and patients. These cells were tested by BD FACSverse (BD Biosciences, San Diego, CA, USA), including T cells (CD45^+^CD3^+^CD19^−^), B cells (CD45^+^CD3^−^CD19^+^), Breg cells (CD45^+^CD3^−^CD19^+^CD1d^+^CD5^+^), DC (CD3^−^CD19^−^

CD56^−^CD14^−^CD11c^+^HLA-DR^+^). The results were analyzed by Flowjo7.6. In addition, we also analyzed and compared the significant differences of Breg cells and DC between genotypes using the SPSS11.0 software.

### SNP Selection and Genotyping

According to previous studies, we selected seven SNPs of IL-35 (including four SNPs of IL-12 p35 and three SNPs of EBI3), including IL-12 p35: rs2243123, rs2227314, rs2243131, rs2243115. EBI3: rs428253, rs9807813, and rs4740. The SNPs of the IL-35 encoding gene were synthesized by the Shanghai Biosune Biotechnology Company. The Hardy-Weinberg equilibrium (HWE) was used to calculate the consistency of all genotype frequencies with all expected control frequencies. Furthermore, information about the SNPs genotypes of IL-12 p35 and EBI3 genes is shown in **Supplementary Table 1** (**Table S1**).

### Enzyme-Linked Immunosorbent Assay

Two milliliters of heparinized venous blood were obtained from patients with uveitis and healthy controls, and their serum was collected after centrifugation by Microcentrifuge (M1324R, RWD LifescienceCo., Ltd, China) at 1500 rpm for 5 min. Subsequently, Human-IL-35 (Cusabio, Cat# CSB-E13126h) ELISA kits were used to evaluate the levels of IL-35 in human serum, according to the manufacturer’s instructions, and were tested using a microplate reader (Bio Tek, Synergy LX, USA). ELISA Calc and Prism 8.0 were used to analyze the concentration levels of IL-35 in the serum of all statisticians. And the concentration of IL-35 in the serum of all recruited subjects (80 cases in total) was analyzed using the SPSS11.0 software to count the mean ± SD.

### Statistical Analysis

We used the Social Science Statistics Package (SPSS) v.11.0 for statistical analysis. The Shapiro–Wilk test was used to test the normality of the data, and the continuous data were described as the mean ± SD of the normally distributed data. The difference of continuous data was evaluated by a *t*-test and a one-way ANOVA test. The difference of categorical data is satisfied by the chi-squared test or Fisher’s exact test. The HWE was used to compare the observed genotype with the expected frequency. The case where the two-sided *p*-value is less than 0.05 is considered to be statistically significant.

## Results

### Characteristics, Clinical Features, and the Immune Status of the Population

**Figure 1:**
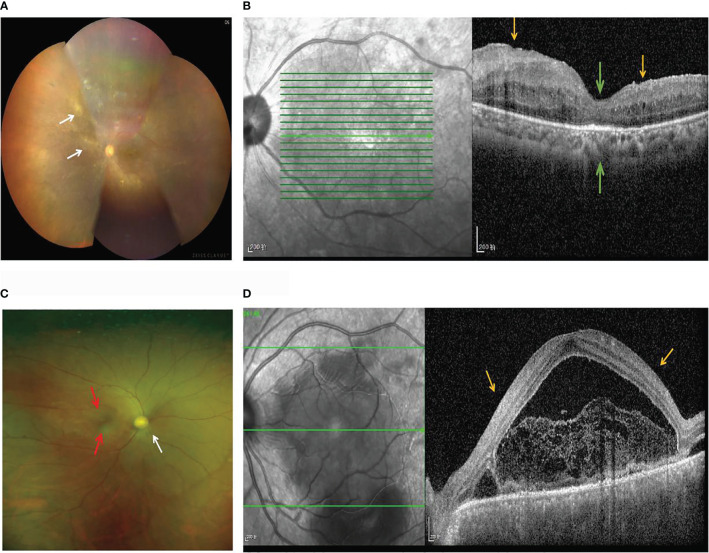
Fundus imaging and OCTA in patients with BD and VKH. **(A)** The color fundus photograph of a patient with BD shows perivenous white sheathing (white arrows). **(B)** OCTA of a patient with BD shows a wedge-shaped localized retinal nerve fiber layer micro-thinning (yellow arrows) in the superior macula and the papillomacular bundle, and the central foveal thinning (green arrows). **(C)** The fundus photograph of a patient with VKH syndrome shows swollen optic disks (white arrows), serous inflammatory infiltration and exudation (red arrows), and an unclear optic disk boundary. **(D)** OCTA of a patient with VKH syndrome shows the presence of subretinal fluid, with multifocal SRDs (yellow arrows).

The basic demographic characteristics of patients with BD and those with VKH syndrome are presented in **Table S2**. There were no significant differences in gender (BD: χ^2^ = 0.272, *p =* 0.602; VKH: χ^2^ = 0.052, *p =* 0.819) and age (BD: *t* = 0.028, *p =* 0.978; VKH: *t* = -1.358, *p =* 0.185) between patients with BD or VKH and healthy controls, shown in **Table S2**. The important clinical manifestations of 32 patients are shown: 90.9% of patients with BD had mouth ulcers, 57.14% of patients with VKH syndrome had headache, 47.62% of patients with VKH syndrome had tinnitus, and 47.42% had alopecia or gray hair (**Table S2**). In the acute phase, the ocular involvement of patients with BD is usually characterized by occlusive retinal vasculitis. The color fundus photograph exhibits perivenous white sheathing (**Figure 1A**). OCTA shows a wedge-shaped localized retinal nerve fiber layer micro-thinning in the superior macula and central foveal thinning (**Figure 1B**). Patients with acute VKH are associated with neurological panuveitis. The fundus photograph shows serious inflammatory infiltration and exudation, swollen optic disks, and unclear optic disk boundary (**Figure 1C**). OCTA shows the presence of subretinal fluid, with multifocal serous retinal detachments (SRDs) (**Figure 1D**).

**Figure 2:**
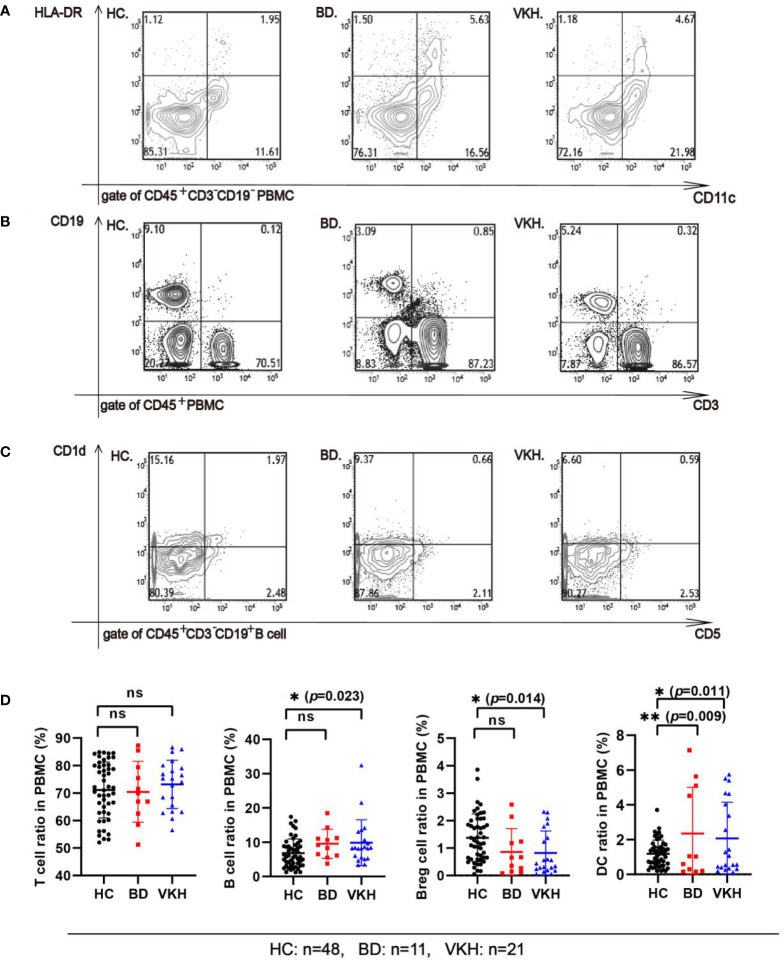
Comparing the subsets of immune cells from the PBMC of the healthy control group and patients. **(A)** The percentage of DC cells in the PBMC of the healthy control group and patients (gate of CD45^+^ cells). Representative flow cytometry plot shows the percentage of CD45^+^CD3^−^CD19^−^CD56^−^CD14^−^CD11c^+^HLA-DR^+^ DC cells in the PBMC of the healthy control group (HC), in BD patient, and in VKH patient. **(B)** The percentage of T and B cells in the PBMC of the healthy control group and patients (gate of CD45^+^ cells). Representative flow cytometry plot shows the percentage of CD45^+^CD3^+^ T cells, CD45^+^CD3^−^CD19^+^B cells in the PBMC of HC, in BD patient, and in VKH patient. **(C)** The percentage of Breg cells (CD45^+^CD3^+^CD19^+^CD1d^+^CD5^+^ B cells) in the PBMC between the healthy control group and patients (gate of CD45^+^CD3^+^CD19^+^ B cells). Representative flow cytometry plot shows the percentage of CD45^+^CD3^−^CD19^+^ CD1d^+^CD5^+^ Breg cells in the PBMC of HC, BD patient, and in VKH patient. **(D)** The percentage of T cell, B cell, Breg cell and DC in PBMC of healthy control group and patients (*p<0.05; **p<0.01; ns, *p*>0.05).

The information about the immune statuses of patients with BD and VKH is provided in **Table S3**. Analysis of the patient’s PBMC immune cells showed that the percentage of DC (the reference range: 0.5%–2.5%) was increased in 36.36% of patients with BD and 38.09% of patients with VKH syndrome (**Figure 2A**). There were 18.18% of patients with BD and 14.28% of patients with VKH syndrome showing an increase in the percentage of T cells (the reference range: 60%–85%) (**Figure 2B**). In addition, we found that in 63.64% of patients with BD and 52.38% of patients with VKH syndrome, the percentage of Breg cells (the reference range: 0.8%–2.2%) was decreased (**Figure 2C**). Overall, in patients with BD or VKH, there were different degrees of immune disorders (**Figure 2D**). Notably, in our study, no matter whether they are patients with BD or those with VKH syndrome, the expression of Breg cells decreased, while the expression of the DC increased in PBMC, which may lead to an imbalance of immune response and inflammation in patients.

### The Serum Levels IL-35 in Patients With BD and VKH

**Figure 3:**
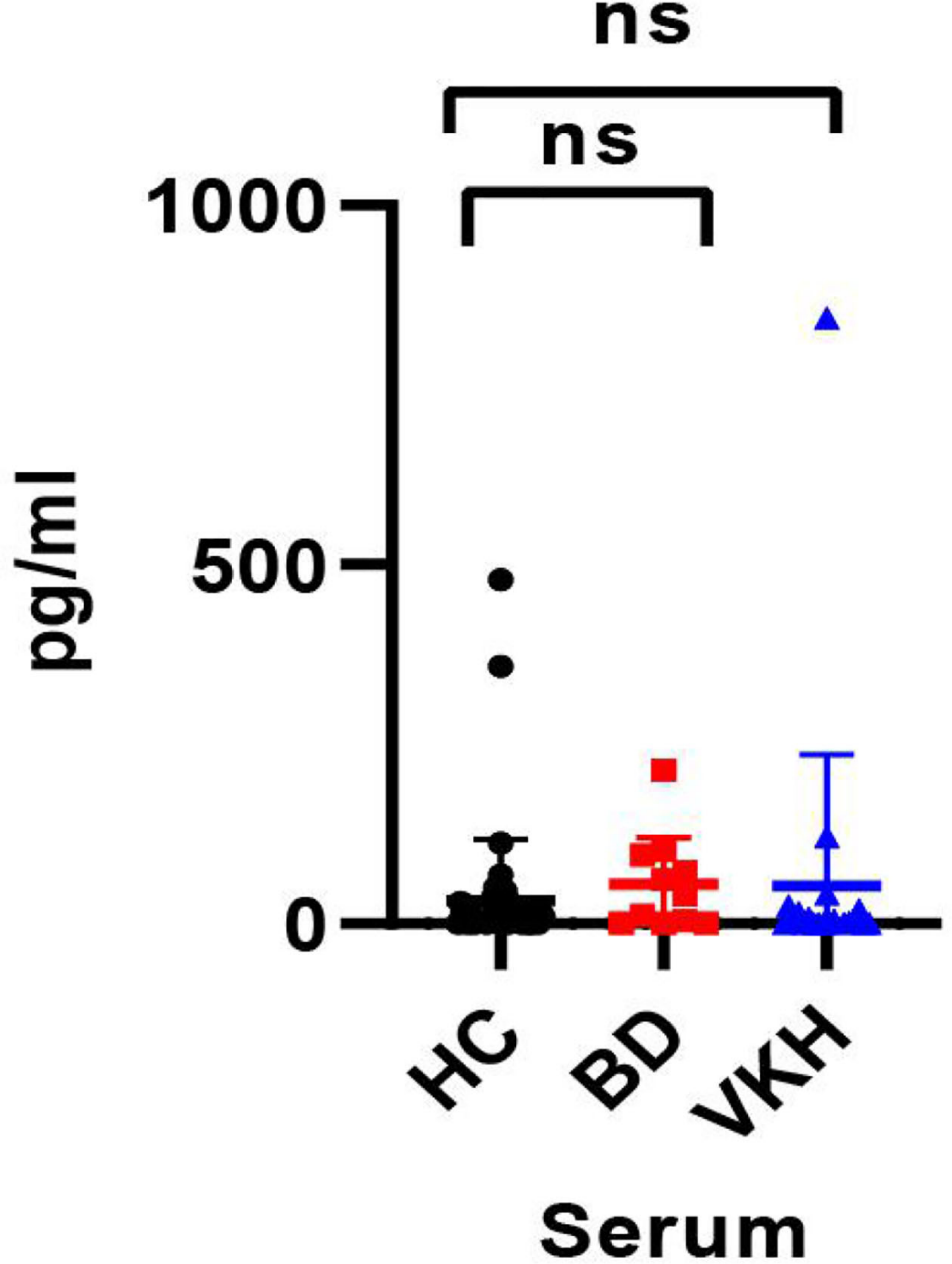
The concentration of IL-35 in serum of the healthy control group and patients with BD or VKH (ns, *p* > 0.05).

The concentration of IL-35 in the serum of all recruited subjects was detected using the human ELISA kit. Compared to healthy controls, the serum IL-35 concentration of patients with BD (32.426 ± 85.294 pg/ml *vs.* 54.749 ± 65.583 pg/ml, *p* = 0.420) was not changed (**Figure 3**). And the serum IL-35 concentration of patients with VKH patients was not different from these of healthy controls (52.266 ± 183.383 pg/ml *vs.* 32.426 ± 85.294 pg/ml, *p* = 0.540) (**Figure 3**).

### Association of IL-35 Gene Polymorphisms With the Risk of Patients With BD and VKH

The allele and genotype frequencies of four SNPs of IL-12 p35 in patients with BD and those with VKH syndrome and healthy controls are presented in **Tables 1**, **2**. There were no significant differences in allele and genotype frequencies of all genotypes IL-12 p35 SNPs (rs2243123, rs2227314, rs2243131, and rs2243115, *p* > 0.05) between patients with BD or VKH patients and healthy controls. However, we analyzed the allele and genotype frequencies of three SNPs of EBI3 (rs428253, rs9807813, and rs4740) in **Tables 3**, **4**, found that the EBI3 rs428253 genotype CC showed a statistical difference between patients with BD and healthy controls (**Table 3**, *p =* 0.021), and the EBI3 rs428253 allele C/G also showed a statistical difference between patients with BD and healthy controls (**Table 3**, *p =* 0.034). But our results could not demonstrate a significant association between the rs9807813 and rs4740 allele and genotype frequencies between patients with BD or VKH, and healthy controls. Thus, there was a significant association between the polymorphism of rs428253 and susceptibility to BD disease in our study.

**Table 1:** IL-12A genotypes and alleles frequencies of genotypes SNPs in BD and healthy controls.

SNPs ID	Genotypes	Patients	Controls	*p* value	*OR*	*95%CI*
rs2243123	TT	9	43	0.473	1.911	0.319-11.450
	CC	0	0	−	−	−
	TC	2	5	0.473	0.523	0.087-3.135
	C	2	5	0.487	0.549	0.099-3.037
	T	20	91	0.487	1.820	0.329-10.060
rs2227314	GG	4	19	0.322	2.293	0.430-12.237
	TT	2	2	0.095	0.196	0.024-1.576
	GT	5	27	0.517	1.543	0.414-5.757
	G	13	65	0.441	1.452	0.561-3.759
	T	9	31	0.441	0.689	0.266-1.784
rs2243131	AA	8	34	0.90	0.911	0.210-3.944
	CC	0	0	−	−	−
	AC	3	14	0.900	1.098	0.254-4.755
	A	19	82	0.909	0.925	0.241-3.543
	C	3	14	0.909	1.081	0.282-4.142
rs2243115	TT	7	36	0.444	1.714	0.426-6.892
	GG	0	0	−	−	−
	TG	4	12	0.444	0.583	0.145-2.345
	G	4	12	0.483	0.643	0.186-2.223
	T	18	84	0.483	1.556	0.450-5.380

Fisher’s Exact Test; OR, odds ratio; CI, confidence interval.

**Table 2:** IL-12A genotypes and alleles frequencies of genotypes SNPs in VKH and healthy controls.

SNPs ID	Genotypes	Patients	Controls	*p* value	*OR*	*95%CI*
rs2243123	TT	15	43	0.058	3.440	0.915-12.934
	CC	0	0	−	−	−
	TC	6	5	0.058	0.291	0.077-1.093
	C	6	5	0.070	0.330	0.095-1.148
	T	36	91	0.070	3.033	0.871-10.566
rs2227314	GG	6	19	0.381	1.638	0.540-4.968
	TT	3	2	0.136	0.261	0.040-1.693
	GT	12	27	0.945	0.964	0.342-2.716
	G	24	65	0.233	1.573	0.746-3.316
	T	18	31	0.233	0.636	0.302-1.341
rs2243131	AA	15	34	0.960	0.971	0.313-3.016
	CC	2	0	0.090	1.105	0.962-1.270
	AC	4	14	0.378	1.750	0.499-6.135
	A	34	82	0.510	1.378	0.530-3.586
	C	8	14	0.510	0.726	0.279-1.888
rs2243115	TT	16	36	0.916	0.938	0.283-3.106
	GG	1	0	0.128	1.050	0.954-1.155
	TG	4	12	0.590	1.417	0.398-5.045
	G	6	12	0.774	0.857	0.298-2.461
	T	36	84	1.000^#^	1.167	0.406-3.350

Fisher’s Exact Test; OR, odds ratio; CI, confidence interval.

**Table 3:** EBI3 genotypes and alleles frequencies of genotypes SNPs in BD and healthy controls.

SNPs ID	Genotypes	Patients	Controls	*p* value	*OR*	*95%CI*
rs428253	GG	0	4	0.321	0.917	0.842-0.998
	CC	9	20	0.021*	0.159	0.031-0.815
	GC	2	24	0.055	4.500	0.879-23.043
	C	20	64	0.034*	0.200	0.044-0.909
	G	2	32	0.034*	5.000	1.100-22.729
rs9807813	CC	6	35	0.233	2.244	0.584-8.627
	TT	0	1	0.629	0.979	0.940-1.020
	CT	5	12	0.177	0.400	0.103-1.550
	C	17	82	0.349	1.723	0.547-5.424
	T	5	14	0.349	0.580	0.184-1.828
rs4740	GG	1	18	0.069	6.000	0.708-50.847
	AA	4	8	0.143	0.350	0.083-1.483
	GA	6	22	0.602	0.705	0.189-2.628
	A	14	38	0.056	0.374	0.143-0.978
	G	8	58	0.056	2.671	1.023-6.977

Fisher’s Exact Test; OR, odds ratio; CI, confidence interval;* there is a significant difference (*p<.0.05).

**Table 4:** EBI3 genotypes and alleles frequencies of genotypes SNPs in VKH and healthy controls.

SNPs ID	Genotypes	Patients	Controls	*p* value	*OR*	*95%CI*
rs428253	GG	0	4	0.173	0.917	0.842-0.998
	CC	11	20	0.410	0.649	0.232-1.820
	GC	10	24	0.856	1.100	0.394-3.070
	C	32	64	0.263	0.625	0.273-1.429
	G	10	32	0.263	1.600	0.700-3.659
rs9807813	CC	15	35	0.899	1.077	0.344-3.370
	TT	1	1	0.542	0.426	0.025-7.145
	CT	5	12	0.916	1.067	0.322-3.534
	C	35	82	0.754	1.171	0.435-3.152
	T	7	14	0.754	0.854	0.317-2.297
rs4740	GG	8	18	0.963	0.975	0.339-2.806
	AA	5	8	0.485	0.640	0.182-2.254
	GA	8	22	0.551	1.375	0.482-3.921
	A	18	38	0.719	0.874	0.419-1.823
	G	24	58	0.719	1.145	0.549-2.388

Fisher’s Exact Test; OR, odds ratio; CI, confidence interval.

### Association of IL-35 Gene Polymorphisms With the Immune Status of Patients With BD and VKH

The associations between allele and genotype frequencies of seven SNPs of IL-35 and the immune statuses of BD and VKH patients are detailed in **Tables 5**, **S4–S9**. There was a statistical difference in allele frequency of the IL-12 p35 rs2243131 between VKH patients with the Breg cell attenuated and those without (*χ^2^* = 6.301, *p =* 0.018, **Table 5**). Although the overall percentage of DC cells in PBMC of BD and VKH patients were higher than the healthy controls. In our study, the SNPs of IL-35 were not related to the expression of DC cells. And there were no significant associations between genotype and allele frequencies of the other SNPs and the immune statuses (including T cell, B cell, the results not shown).

**Table 5:** Associations of IL-12p35 (rs2243131) with immune states in BD or VKH patients.

Diseases	Immune states	↑/↓	Genotypes			*x* ^2^	*p* value	Allele		*x* ^2^	*p* value
			AA	AC	CC			A	C		
BD	Breg	↑	2	2	0	1.637	0.491	6	2	1.378	0.527
		↓	6	1	0			13	1		
	DC	↑	2	2	0	1.637	0.491	6	2	1.378	0.527
		↓	6	1	0			13	1		
VKH	Breg	↑	5	3	2	4.630	0.099	13	7	6.301	0.018*
		↓	10	1	0			21	1		
	DC	↑	5	2	1	0.505	0.777	12	4	0.594	0.454
		↓	10	2	1			22	4		

↑, cell expression increased or in the normal range; ↓, cell expression decreased or in the normal range; Fisher’s Exact Test; *There is a statistical difference (*p<0.05).

### Association of IL-35 Gene Polymorphisms With the Clinical Features of Patients With BD and VKH

The associations between allele and genotype frequencies of seven SNPs of IL-35 with the clinical features of patients with BD and VKH are provided in **Tables 6**, **S10–S15**. There was a statistical difference in allele frequency of the EBI3 rs4740 between VKH patients with headache and without it (*χ^2^* = 7.291, *p =* 0.012, **Table 6**). And this allele gene frequency in patients with VKH syndrome was also a statistical difference between the tinnitus and those without (*χ^2^* = 4.972, *p =* 0.033, **Table 6**). However, there were no significant associations between all genotype and allele frequencies of SNPs and the clinical features of patients with BD.

**Table 6:** Associations of EBI3 (rs4740) with clinical manifestations in BD or VKH patients.

Diseases	Manifestations	+/−	Genotypes			*x* ^2^	*p* value	Allele		*x* ^2^	*p* value
			GG	GA	AA			A	G		
BD	Mouth ulcers	+	1	6	3	2.554	0.455	12	8	1.257	0.515
		−	0	0	1			2	0		
VKH	Headache	+	7	4	1	5.722	0.053	6	18	7.292	0.012*
		−	1	4	4			12	6		
	Tinnitus	+	6	3	1	3.990	0.166	5	15	4.972	0.033*
		−	2	5	4			13	9		
	Alopecia/Grey hair	+	5	3	2	1.218	0.641	7	13	1.123	0.289
		−	3	5	3			16	16		

+, with symptom; -, without symptom; Fisher’s Exact Test; *There is a statistical difference (*p<0.05).

### Association of Serum IL-35 Levels With IL-35 Genotypes of Patients With BD and VKH

We evaluated the association between serum levels and SNP of IL-35 in patients with BD and VKH (**Tables 7**, **8**). The results revealed that there was an association between the frequency of the IL-12 p35 rs2243115 genotype and the human serum IL-35 levels in patients with VKH syndrome (*χ^2^* = 7.291, *p =* 0.012, **Table 8**). The concentration of serum IL-35 in patients with VKH syndrome was no significantly different from that in healthy controls. There was no correlation between other SNPs and serum levels of IL-35.

**Table 7:** Associations of serum IL-35 levels with IL-35 genotypes in BD patients.

SNP ID	Genotypes	n	IL-35 Level (pg/ml)	*F/t*	*p* value
rs2243123	TT	9	48.719 ± 71.055	-0.627	0.546^@^
	TC	2	81.886 ± 28.642		
	CC	0	–		
rs2227314	GG	4	55.131 ± 36.516	0.330	0.895
	GT	5	75.492 ± 88.477		
	TT	2	2.128 ± 2.868		
rs2243131	AA	8	73.736 ± 66.851	1.836	0.100^@^
	AC	3	1.452 ± 2.342		
	CC	0	–		
rs2243115	GG	0	–	0.010	0.992^@^
	TG	4	54.461 ± 106.035		
	TT	7	54.914 ± 39.328		
rs428253	GG	0	–	0.549	0.596^@^
	GC	2	30.867 ± 43.511		
	CC	9	60.056 ± 70.466		
rs9807813	CC	6	58.916 ± 84.302	-0.220	0.831^@^
	CT	5	49.749 ± 42.566		
	TT	0	–		
rs4740	GG	1	39.841	1.523	0.456
	GA	6	67.459 ± 82.599		
	AA	4	39.411 ± 47.488		

^@^t test; others data analyzed used one-way ANOVA.

**Table 8:** Associations of serum IL-35 levels with IL-35 genotypes in VKH patients.

SNP ID	Genotypes	n	IL-35 Level (pg/ml)	*F/t*	*p* value
rs2243123	TT	15	15.269 ± 31.241	-0.925	0.397^@^
	TC	6	144.759 ± 342.344		
	CC	0	–		
rs2227314	GG	6	7.769 ± 8.524	1.134	0.423
	GT	12	83.841 ± 241.61		
	TT	3	14.965 ± 22.325		
rs2243131	AA	15	70.311 ± 215.983	0.896	0.567
	AC	2	20.369 ± 28.664		
	CC	4	0.548 ± 0.896		
rs2243115	GG	1	4.157	3.526	0.030*
	TG	4	33.885 ± 58.228		
	TT	16	59.868 ± 209.246		
rs428253	GG	0	–	0.960	0.349^@^
	GC	10	92.617 ± 264.081		
	CC	11	15.584 ± 35.765		
rs9807813	CC	15	14.364 ± 31.795	2.529	0.080
	CT	5	174.684 ± 373.829		
	TT	1	8.708		
rs4740	GG	8	24.306 ± 41.809	0.561	0.812
	GA	8	3.940 ± 5.859		
	AA	5	174.326 ± 374.037		

^@^t test; others data analyzed used one-way ANOVA; *There is a statistical difference (*p<0.05).

## Discussion

The disorder of immune tolerance has been considered a vital cause for the pathogenesis of autoimmune diseases. Participation in the induction of immune tolerance and the loss of cells with negative immune-regulation functions are essential factors in the occurrence and development of AIU. Breg cells play a negative immune-regulation role in autoimmune diseases, the absence of Breg cells may be an essential reason for the occurrence of some diseases, and they are decreased and/or functionally impaired in these autoimmune diseases, including EAE, rheumatoid arthritis (RA), and SLE (13, 18). However, the status of Breg cells in patients with uveitis is still not known. Our study showed that the percentage of Breg cells is decreased in the blood, suggesting that the lack of negative immune-regulation of Breg cells may be an essential reason for the imbalance of immune response in uveitis. The decrease of Breg cells may contribute to the immune disorders and inflammation in uveitis.

There were no reports about the relationship between the SNPs of IL-35 and the pathogenesis of uveitis (31). We have analyzed the relationship between IL-35 gene SNPs and genetic susceptibility in the Chinese Han population with uveitis (mainly BD and VKH syndrome). Our results suggest that the IL-12 p35 rs2243131 A/C allele was related to the disorder of Breg cells in VKH patients, and IL-35 and its subunit IL-12p35 preferentially induced the expansion of Breg and Treg cells, inhibited the activation of DC, and inhibited the expansion of pathogenic Th17 and Th1 cells (13, 14, 32), to slow down the development of autoimmune diseases. Thus, the IL-12 p35 A/C allele mutation may be the key factor of Breg cells reduction in VKH patients. Additionally, there was no correlation between other immune cells and IL-35 gene polymorphism, but it cannot eliminate these sites associated with the function of immune cells.

VKH patients are usually with extra-ocular manifestations such as headache, hearing loss (7). In our study results, 57.14% of VKH patients had a headache, and 47.62% had tinnitus. And there was associations between the polymorphisms of the EBI3 rs4740 and the occurrence of headache or tinnitus in VKH patients. Previous studies reported that there were associations between EBI3 rs4740 polymorphism and SLE, UC (24, 33). Thus, the polymorphism of the EBI3 rs4740 may be correlated with the occurrence of autoimmune disease.

We also measured the concentration of IL-35 in the serum of all subjects. Compared with the healthy controls group, the concentration of serum IL-35 in patients with BD was not changed, which is different from the previous study about active BD or BD patients with only mucocutaneous involvement (34, 35), but is consistent with the result of BD patients with mucocutaneous manifestations plus ocular involvement. Moreover, the serum IL-35 concentration of VKH patients was not difference from these of healthy controls, which is consistent with the previous study about inactive VKH patients (36). Thus, the disease statuses, and ethnic differences of patients may be important factors for the level of IL-35. Moreover, IL-35 SNPs were associated with RA, type I diabetes, SLE, and other autoimmune diseases (24, 37, 38). Some studies have explored the relationship between IL-35 (including IL-12 p35 and EBI3) gene SNPs and susceptibility to autoimmune diseases. For instance, IL-12 p35 rs2243115 and rs568408 were novel genetic risk factors for Alzheimer’s disease in the Han Chinese population (39). IL-12 p35 rs2243115 was significantly related to the high RA disease risk in a Chinese population (37). EBI3 rs428253 had a related effect on Chinese Han patients with allergic rhinitis (40). EBI3 rs4740 had a significant correlation with the clinical manifestations of Chinese Han patients with SLE (24). All of this evidence shows that IL-35 is involved in the occurrence and progression of autoimmune diseases. Unlike previous reports, we have shown that the EBI3 rs428253 CC genotype and C/G allele may be a susceptibility genes for BD patients. Nevertheless, the role of IL-35 SNPs in the pathogenesis of BD or VKH syndrome and the regulation of the immune response still needs further studying. Additionally, large samples would help determine the correlation between SNPs and diseases.

In conclusion, our research mainly analyzed the association between IL-35 SNPs and AIU disease susceptibility, immune cells, clinical symptoms, and serum levels. In our study, the polymorphism of EBI3 rs4740, which at the coding region was associated with the occurrence of clinical manifestations in VKH patients. Other SNPs are at the non-coding region. SNPs in the non-coding region not only change gene regulation, but also affect gene expression by affecting gene splicing, binding with transcription factors, degradation of messenger RNA, or other ways. Thus, the polymorphism of IL-35 SNPs are involved in the occurrence of AIU. Above all, uveitis may be affected by genetic and immune factors. Uveitis is resulting from the interaction between genetic and various factors, which may provide a new basis for its diagnosis and treatment.

## Data Availability Statement

The original contributions presented in the study are publicly available. This data can be found in dbSNP, batch ID: 1063306.

## Ethics Statement

The studies involving human participants were reviewed and approved by Committee of Shandong Eye Hospital, Shandong Eye Institute, Shandong First Medical University and Shandong Academy of Medical School (2019-G-012) granted ethical approval for the study. Written informed consent to participate in this study was provided by the participants’ legal guardian/next of kin. Written informed consent was obtained from the individual(s) for the publication of any potentially identifiable images or data included in this article.

## Author Contributions

WL, TTL, MF, and SZ designed research, planned and performed experiments, and drafted and revised the manuscript. TL, YY, QS, XL, MZ, and XX organized the data, generated the figures and tables. TTL provided clinical samples. All authors contributed to the article and approved the submitted version.

## Funding

This work was supported by grants from the Natural Science Foundation of China (81500710), the Shandong Key Research and Development Project (2019GSF108189), projects of medical and health technology development program in Shandong province (2015WS0194 and 2019WS186), and the Innovation Project of Shandong Academy of Medical Sciences.

## Conflict of Interest

The authors declare that the research was conducted in the absence of any commercial or financial relationships that could be construed as a potential conflict of interest.

## Publisher’s Note

All claims expressed in this article are solely those of the authors and do not necessarily represent those of their affiliated organizations, or those of the publisher, the editors and the reviewers. Any product that may be evaluated in this article, or claim that may be made by its manufacturer, is not guaranteed or endorsed by the publisher.
