# Supplementary material for: Association Between Interleukin 35 Gene Single Nucleotide Polymorphisms and the Uveitis Immune Status in a Chinese Han Population
Source: Front Immunol. 2021 Dec 7;12:758554. doi: 10.3389/fimmu.2021.758554 (PMC8688856; doi:10.3389/fimmu.2021.758554)
Supplement: Supplementary file 1 [file DataSheet_1.pdf]

Supplementary Material

Association between Interleukin 35 Gene Single Nucleotide Polymorphisms and the Uveitis Immune Status in a Chinese Han Population

Meng Feng [1*], Shuping Zhou [1*], Tong Liu [2], Yong Yu [1], Qinghong Su [1], Xiaofan Li [1], Min Zhang [2], Xiao Xie [3,4,5], Tingting Liu [3,4,6#], Wei Lin[1#].

1. School of Basic Medicine, Shandong First Medical University&Shandong Academy of Medical Sciences, Jinan 250002, China.
2. Departments of Medicine, Tibet Nationalities University, Xianyang, China.
3. Eye Hospital of Shandong First Medical University (Shandong Eye Hospital), Jinan, China.
4. State Key Laboratory Cultivation Base, Shandong Provincial Key Laboratory of Ophthalmology, Shandong Eye Institute, Shandong First Medical University & Shandong Academy of Medical Sciences, Qingdao, China.
5. Shandong University of Traditional Chinese Medicine, Jinan, China.
6. School of Ophthalmology, Shandong First Medical University, Jinan, China.

[#]Co-corresponding Author:
Wei Lin, Ph.D.
School of Basic Medicine, Shandong First Medical University&Shandong Academy of Medical Sciences, Jinan 250002, China.
Phone: (+86)-0531-82979938,
Email: linw1978@163.com or weilin11@fudan.edu.cn.
Tingting Liu, Ph,D.
Eye Hospital of Shandong First Medical University (Shandong Eye Hospital), Jinan, China; State Key Laboratory Cultivation Base, Shandong Provincial Key Laboratory of Ophthalmology, Shandong Eye Institute, Qingdao, China; Shandong First Medical University & Shandong Academy of Medical Sciences, Qingdao, China; School of Ophthalmology, Shandong First Medical University, Jinan, China.
Email: tingtingliu@vip.sina.com.

[*] Co-first author.

Table S1: SNPs genotyped in IL-12p35 and EBI3 genes.

Gene	SNPs ID	Chr	Chr position	Allele	mRNA	Region	x^2	*p* value for H-W test
IL-12p35	rs2243123	3	159709651	T/C	NM_000882.3	Intron2	0.145	0.930
IL-12p35	rs2227314	3	159712054	G/T	NM_000882.3	Intron6	3.936	0.139
IL-12p35	rs2243131	3	159712058	A/C	NM_000882.3	Intron6	1.399	0.497
IL-12p35	rs2243115	3	159706280	T/G	NM_000882.3	5'Flanking	0.979	0.613
EBI3	rs428253	19	4229913	G/C	NM_005755.2	Intron1	0.548	0.760
EBI3	rs9807813	19	4232415	C/T	NM_005755.2	Intron2	0.001	0.999
EBI3	rs4740	19	4236996	G/A	NM_005755.2	Exon5	0.084	0.959

Table S2: Demographic characteristics and clinical features of Uveites patients and healthy controls.

Clinical information	BD	VKH	Healthy Controls	x^2/t	*P* value
Male	5 (45.5%)	12 (57.14%)	26 (54.17%)	0.272*(BD)	0.602
Female	6 (54.5%)	9 (42.86%)	22 (45.83%)	0.052*(VKH)	0.819
Age	41.91±20.08		42.08±12.25	0.028**(BD)	0.978
		47.86±17.71		-1.358**(VKH)	0.185
Mouth ulcers	10 (90.9%)				
Headache		12 (57.14%)			
Tinnitus		10 (47.62%)			
Alopecia / Grey hair		10 (47.62%)			

* x^2 test.; ** *t*-test.

Table S3: Information about the BD (11 patients) and VKH (21 patients) patients' immune states.

		BD	VKH
T cell	↑	2 (18.18 %)	3 (14.28 %)
	↓	2 (18.18 %)	1 (4.76 %)
B cell	↑	0	2 (9.52 %)
	↓	1(9.09 %)	3 (14.28 %)
DC cell	↑	4 (36.36 %)	8 (38.09 %)
	↓	4 (36.36 %)	7 (33.33 %)
Breg cell	↑	1 (9.09 %)	2 (9.52 %)
	↓	7 (63.64 %)	11 (52.38 %)

Table S4: Associations of IL-12p35 (rs2243123) with immune states in BD or VKH patients

Diseases	Immnune states	↑/↓	Genotypes			x^2	*p* value	Allele		x^2	*p*value
			TT	TC	CC			C	T		
BD	Breg	↑	3	1	0	0.196	1.000#	1	7	0.177	1.000#
		↓	6	1	0			1	13		
	DC	↑	3	1	0	0.196	1.000#	1	7	0.177	1.000#
		↓	6	1	0			1	13		
VKH	Breg	↑	7	3	0	0.019	1.000#	3	17	0.016	1.000#
		↓	8	3	0			3	19		
	DC	↑	6	2	0	0.081	1.000#	2	14	0.067	1.000#
		↓	9	4	0			4	22		

↑, cell expression increased or in the normal range; ↓, cell expression decreased or in the normal range; Fisher's Exact Test; #Corrected *p* value was 1.000.

Table S5: Associations of IL-12p35 (rs2227314) with immune states in BD or VKH patients

Diseases	Immnune states	↑/↓	Genotypes			x^2	*p* value	Allele		x^2	*p*value
			GG	GT	TT			G	T		
BD	Breg	↑	1	2	1	0.413	0.814	4	4	0.430	0.662
		↓	3	3	1			9	5		
	DC	↑	0	3	1	3.654	0.161	3	5	2.424	0.187
		↓	4	2	1			10	4		
VKH	Breg	↑	2	6	2	0.955	0.620	10	10	0.795	0.372
		↓	4	6	1			14	8		
	DC	↑	3	3	2	2.272	0.321	9	7	0.008	0.927
		↓	3	9	1			15	11		

↑, cell expression increased or in the normal range; ↓, cell expression decreased or in the normal range; Fisher's Exact Test.

Table S6: Associations of IL-12A (rs2243115) with immune states in BD or VKH patients

Diseases	Immnune states	↑/↓	Genotypes			x^2	*p* value	Allele		x^2	*p value*
			GG	TG	TT			G	T		
BD	Breg	↑	0	1	3	0.351	1.000#	1	7	0.273	1.000#
		↓	0	3	4			3	11		
	DC	↑	0	2	2	0.505	0.576	2	6	0.393	0.602
		↓	0	2	5			2	12		
VKH	Breg	↑	0	2	8	0.955	0.620	2	18	0.573	0.665
		↓	1	2	8			4	18		
	DC	↑	1	1	6	1.918	0.383	3	13	0.421	0.658
		↓	0	3	10			3	23		

↑, cell expression increased or in the normal range; ↓, cell expression decreased or in the normal range; Fisher's Exact Test; #Corrected *p* value was still 1.000.

Table S7: Associations of EBI3 (rs428253) with immune states in BD or VKH patients

Diseases	Immnune states	↑/↓	Genotypes			x^2	*p* value	Allele		x^2	*p value*
			GG	GC	CC			C	G		
BD	Breg	↑	0	1	3	0.196	1.000#	7	1	0.177	1.000#
		↓	0	1	6			13	1		
	DC	↑	0	1	3	0.196	1.000#	7	1	0.177	1.000#
		↓	0	1	6			13	1		
VKH	Breg	↑	0	3	7	2.376	0.123	17	3	1.633	0.201
		↓	0	7	4			15	7		
	DC	↑	0	3	5	0.531	0,659	13	3	0.365	0.546
		↓	0	7	6			19	7		

↑, cell expression increased or in the normal range; ↓, cell expression decreased or in the normal range; Fisher's Exact Test; #Corrected *p* value still 1.000;

Table S8: Associations of EBI3 (rs9807813) with immune states in BD or VKH patients

Diseases	Immnune states	↑/↓	Genotypes			x^2	*p* value	Allele		x^2	*p*value
			CC	CT	TT			C	T		
BD	Breg	↑	3	1	0	1.061	0.545	7	1	0.749	0.613
		↓	3	4	0			10	4		
	DC	↑	2	2	0	0.052	1.000#	6	2	0.037	1.000#
		↓	4	3	0			11	3		
VKH	Breg	↑	8	2	0	1.222	0.543	18	2	1.222	0.414
		↓	7	3	1			17	5		
	DC	↑	6	2	0	0.646	0.724	14	2	0.323	0.690
		↓	9	3	1			21	5		

↑, cell expression increased or in the normal range; ↓, cell expression decreased or in the normal range; Fisher's Exact Test; #Corrected *p* value still 1.000.

Table S9: Associations of EBI3 (rs4740) with immune states in BD or VKH patients

Diseases	Immnune states	↑/↓	Genotypes			x^2	*p* value	Allele		x^2	*p*value
			GG	GA	AA			A	G		
BD	Breg	↑	1	2	1	1.977	0.368	4	4	1.010	0.315
		↓	0	4	3			10	4		
	DC	↑	1	3	0	4.518	0.104	3	5	3.711	0.054
		↓	0	3	4			11	3		
VKH	Breg	↑	5	4	1	2.258	0.323	6	14	2.577	0.108
		↓	3	4	4			12	10		
	DC	↑	2	4	2	1.070	0.586	8	8	0,538	0.463
		↓	6	4	3			10	16		

↑, cell expression increased or in the normal range; ↓, cell expression decreased or in the normal range; Fisher's Exact Test.

Table S10: Associations of IL-12p35 (rs2243123) with clinical manifestations in BD or VKH patients

Diseases	Manifestations	+/−	Genotypes			x^2	p value	Allele		x^2	$p$value
			TT	TC	CC			C	T		
BD	Mouth ulcers	+	8	2	0	0.244	1.000#	2	18	0.220	1.000#
		−	1	0	0			0	2		
VKH	Headache	+	9	3	0	0.175	1.000#	3	21	0.146	1.000#
		−	6	3	0			3	15		
	Tinnitus	+	9	1	0	0.072	1.000#	1	19	2.689	0.187
		−	6	5	0			5	17		
	Alopecia / Grey hair	+	8	2	0	0.687	0.635	2	18	0.573	0.665
		−	7	4	0			4	18		

+, with symptom; -, without symptom; Fisher's Exact Test; #Corrected p value still 1.000.

Table S11: Associations of IL-12p35 (rs2227314) with clinical manifestations in BD or VKH patients

Diseases	Manifestations	+/−	Genotypes			x^2	p value	Allele		x^2	$p$value
			GG	GT	TT			G	T		
BD	Mouth ulcers	+	4	4	2	1.597	1.000#	12	8	0.075	1.000#
		−	0	1	0			1	1		
VKH	Headache	+	3	7	2	1.428	0.650	13	11	0.667	0.414
		−	3	6	0			12	6		
	Tinnitus	+	3	5	2	0.780	0.841	11	9	0.072	0.789
		−	3	7	1			13	9		
	Alopecia / Grey hair	+	4	5	1	1.355	0.591	13	7	0.963	0.327
		−	2	7	2			11	11		

+, with symptom; -, without symptom; Fisher's Exact Test; #Corrected p value still 1.000.

Table S12: Associations of IL-12p35 (rs2243131) with clinical manifestations in BD or VKH patients

Diseases	Manifestations	+/−	Genotypes			x^2	p value	Allele		x^2	$p$value
			AA	AC	CC			A	C		
BD	Mouth ulcers	+	7	3	0	0.413	1.000#	17	3	0.347	1.000#
		−	1	0	0			2	0		
VKH	Headache	+	9	1	2	3.238	0.328	19	5	0.116	1.000#
		−	6	3	0			15	3		
	Tinnitus	+	7	1	2	2.594	0.320	15	5	0.877	0.445
		−	8	3	0			19	3		
	Alopecia / Grey hair	+	7	2	1	0.396	1.000#	16	4	0.022	1.000#
		−	8	2	1			18	4		

+, with symptom; -,without symptom; Fisher's Exact Test; #Corrected p value still 1.000.

.

Table S13: Associations of IL-12p35 (rs2243115) with clinical manifestations in BD or VKH patients

Diseases	Manifestations	+/−	Genotypes			x^2	*p* value	Allele		x^2	*p*value
			GG	TG	TT			G	T		
BD	Mouth ulcers	+	0	3	7	1.925	0.364	3	17	1.497	0.338
		−	0	1	0			1	1		
VKH	Headache	+	1	3	8	1.508	0.765	5	19	1.961	0.214
		−	0	1	8			1	17		
	Tinnitus	+	1	3	6	2.785	0.188	5	15	3.580	0.087
		−	0	1	10			1	21		
	Alopecia / Grey hair	+	0	3	7	1.231	1.000#	3	17	0.076	1.000#
		−	1	2	8			4	18		

+,with symptom; -, without symptom; Fisher's Exact Test; #Corrected *p* value still 1.000.

Table S14: Associations of EBI3 (rs428253) with clinical manifestations in BD or VKH patients

Diseases	Manifestations	+/−	Genotypes			x^2	*p* value	Allele		x^2	*p*value
			GG	GC	CC			C	G		
BD	Mouth ulcers	+	0	2	8	0.244	1.000#	18	2	0.220	1.000#
		−	0	0	1			2	0		
VKH	Headache	+	0	7	5	1.289	0.387	17	7	0.886	0.347
		−	0	3	6			15	3		
	Tinnitus	+	0	6	4	1.173	0.279	14	6	0.807	0.369
		−	0	4	7			18	4		
	Alopecia / Grey hair	+	0	4	6	0.444	0.505	16	4	0.305	0.580
		−	0	6	5			16	6		

+, with symptom; -, without symptom; Fisher's Exact Test; #Corrected *p* value still 1.000.

Table S15: Associations of EBI3 (rs9807813) with clinical manifestations in BD or VKH patients

Diseases	Manifestations	+/−	Genotypes			x^2	*p* value	Allele		x^2	*p*value
			CC	CT	TT			C	T		
BD	Mouth ulcers	+	6	4	0	1.320	0.455	16	4	0.932	0.411
		−	0	1	0			1	1		
VKH	Headache	+	10	2	0	2.427	0.216	22	2	2.800	0.118
		−	5	3	1			13	5		
	Tinnitus	+	8	2	0	1.231	1.000#	18	2	1,222	0.414
		−	7	3	1			17	5		
	Alopecia / Grey hair	+	8	2	0	1.231	1.000#	18	2	1.222	0.414
		−	7	3	1			17	5		

+, with symptom; -, without symptom; Fisher's Exact Test; #Corrected *p* value still 1.000.
